# Atypical X-linked agammaglobulinemia diagnosed in adulthood with arthritis caused by a hypomorphic BTK splice-site variant: a case report and literature review

**DOI:** 10.3389/fimmu.2026.1849090

**Published:** 2026-05-21

**Authors:** Asuka Takayama, Kazuyuki Meguro, Masaya Yokota, Takahiro Sugiyama, Shunjiro Kurihara, Masaki Hiraguri, Hirokazu Kanegane, Hiroshi Nakajima

**Affiliations:** 1 Department of Allergy and Clinical Immunology, Graduate School of Medicine, Chiba University, Chiba, Japan; 2 Clinical Genetics, Chiba University Hospital, Chiba, Japan; 3 Department of Rheumatology and Allergology Medicine, Japanese Red Cross Narita Hospital, Chiba, Japan; 4 Department of Child Health and Development, Graduate School of Medical and Dental Sciences, Institute of Science Tokyo, Tokyo, Japan

**Keywords:** arthritis, BTK, inborn errors of immunity (IEI), infection, splice-site variant, X-linked agammaglobulinemia (XLA)

## Abstract

X-linked agammaglobulinemia (XLA) is an inborn error of immunity (IEI) caused by loss-of-function variants in the *Bruton tyrosine kinase* (*BTK*) gene, classically presenting in early childhood with recurrent bacterial infections. Here, we report an atypical case of XLA diagnosed in adulthood during evaluation of inflammatory arthritis. A 42-year-old man with no history of severe or recurrent infections initially presented with monoarticular inflammation of the right elbow joint and later developed Doppler-positive synovitis and tenosynovitis of the hands, together with a methicillin-sensitive Staphylococcus aureus lung abscess and a subsequent deep gluteal abscess. Immunological evaluation showed profound hypogammaglobulinemia with markedly reduced circulating B cells. Genetic testing revealed a *BTK* splice-site variant, c.1103-2A>G (NM_000061.3). Functional analyses demonstrated abnormal splicing at the exon 12–13 junction, reduced *BTK* mRNA expression, and reduced but detectable BTK protein expression in monocytes, supporting residual protein expression consistent with a hypomorphic or leaky phenotype. Although XLA is generally considered to show limited genotype-phenotype correlation, residual BTK expression in this case could have contributed, at least in part, to the relatively mild early infectious phenotype. In addition, the patient’s longstanding social withdrawal, with presumably limited exposure to infectious sources, may also have contributed to delayed clinical recognition until adulthood. Importantly, immunoglobulin replacement therapy was followed by sustained prevention of further deep infections and gradual improvement of synovitis, tenosynovitis, and skin manifestations. This case highlights that common rheumatologic presentations in adults, such as arthritis, may mask an underlying IEI. Early recognition of IEI can enable timely immunoglobulin replacement therapy, which may restore humoral immune protection, prevent further severe infections, and improve rheumatologic manifestations. It also underscores the importance of integrating immunological and genetic evaluation with protein- and transcript-level analyses when interpreting atypical presentations and splice-site variants.

## 1 Introduction

Inborn errors of immunity (IEIs), formerly referred to as primary immunodeficiency diseases, comprise a heterogeneous group of genetic disorders caused by germline variants affecting immune development, function, or regulation (1). IEIs are increasingly recognized not only for their susceptibility to infections but also for their association with immune dysregulation, including autoimmunity, autoinflammation, allergy, and malignancy (1). With advances in genetic studies, the clinical spectrum of IEIs has expanded substantially, and delayed or atypical presentations in adulthood are now increasingly appreciated (2).

X-linked agammaglobulinemia (XLA) represents a landmark disease in the history of IEIs. First described in 1952 by Ogden C. Bruton (3), XLA was the first immunodeficiency disorder in which a laboratory abnormality directly guided effective therapy, namely immunoglobulin replacement therapy (IgRT), establishing a paradigm for modern clinical immunology. This seminal observation not only defined XLA as a distinct clinical entity but also laid the foundation for the diagnostic and therapeutic framework of IEIs.

XLA is caused by loss-of-function variants in the *Bruton tyrosine kinase* (*BTK*) gene, which is essential for B-cell development and maturation. Defective BTK signaling results in an early block in B-cell differentiation, leading to a profound deficiency of peripheral B cells and markedly reduced levels of all immunoglobulin isotypes (4). Clinically affected individuals typically present in early childhood, after the waning of maternally derived antibodies, with recurrent bacterial infections involving the respiratory tract, skin, and gastrointestinal system. With the advent of IgRT, most patients with XLA now survive into adulthood (5); however, morbidity related to infections and immune dysregulation remains a significant concern (5, 6).

Although XLA is classically regarded as a pediatric-onset IEI, adult-onset or adult-diagnosed XLA has been repeatedly documented, particularly in patients with atypical or attenuated clinical phenotypes (2, 7–12). In these cases, the absence of a striking history of severe childhood infections often results in substantial diagnostic delay, with diagnosis occurring in adolescence or adulthood (7–11).

Arthritis, both infectious and non-infectious, is now recognized as a relatively common complication in patients with XLA (10, 12–16). Cohort studies have demonstrated that a substantial proportion of patients develop articular involvement, and notably, joint symptoms may precede the diagnosis of XLA in a significant subset of cases (12). The articular manifestations vary widely, encompassing oligoarthritis and polyarthritis of both large and small joints; erosive changes are notably absent in many cases (12, 13). While infectious arthritis has been reported, many cases exhibit sterile inflammatory features, raising questions regarding the underlying pathophysiological mechanisms (13–15).

The etiology of arthritis in XLA remains incompletely understood (12–14). Proposed mechanisms include chronic infection-driven inflammation (13, 16), immune complex–mediated processes (13, 17), and dysregulated innate immune responses in the setting of antibody deficiency (17, 18). Importantly, several studies have reported improvement of arthritis following initiation of IgRT (12, 14, 15), suggesting that restoration of humoral immunity plays a critical role in disease control (12).

Here, we report a case of atypical XLA diagnosed in adulthood in a patient who presented with inflammatory musculoskeletal manifestations. We identified a *BTK* splice-site variant, c.1103-2A>G, and demonstrated aberrant splicing, reduced *BTK* mRNA expression, and residual BTK protein expression, consistent with a hypomorphic or leaky phenotype. Following IgRT, the patient showed gradual improvement of musculoskeletal and cutaneous manifestations, with no recurrence of deep infections during follow-up. This case emphasizes that IEIs may underlie common rheumatologic presentations in adults and highlights the value of detailed genetic, protein, and transcript-level analyses for atypical cases.

## 2 Case presentation

**Figure 1:**
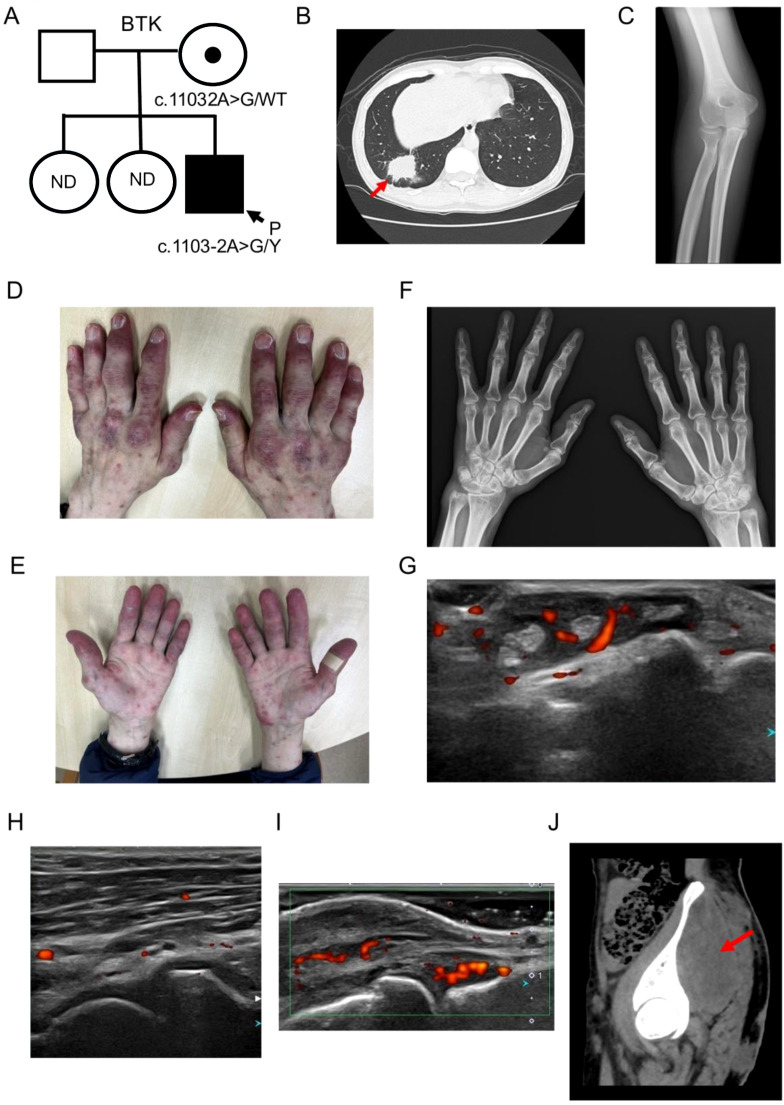
Pedigree and clinical manifestations of the affected patient. **(A)** Pedigree of the affected family. The filled square indicates the affected proband (P). The circle with a central dot indicates an obligate carrier. Squares, males; circles, females; WT, wild-type; Y, hemizygous on the X chromosome, ND; no data. **(B)** Chest CT showing a mass lesion in the right lower lobe (red arrow), consistent with a pulmonary abscess. **(C)** Plain radiograph of the right elbow obtained at the time of rheumatology referral, showing no definitive joint destruction. **(D, E)** Cutaneous and articular findings of the patient’s hands (dorsal **(D)** and palmar **(E)** views) showing erythematous plaques over the extensor aspects of the fingers and scattered palmar lesions. **(F)** Plain radiographs of both hands, showing soft tissue swelling of the proximal interphalangeal joints without definite bony destruction. **(G)** Musculoskeletal ultrasonography of the right wrist demonstrating severe tenosynovitis of the fourth extensor compartment. **(H)** Musculoskeletal ultrasonography of the right elbow showing mild synovial hypertrophy at the brachioradial joint. **(I)** Musculoskeletal ultrasonography of right index MCP joint demonstrating moderate synovial hypertrophy with a moderate Doppler signal and extensor peritendinous inflammation. **(J)** Pelvic CT demonstrating a deep gluteal abscess with extension into the gluteal muscle (red arrow).

The patient was a 42-year-old man with no apparent history of severe or recurrent childhood infections, aside from a single episode of otitis media. There was no family history suggestive of immunodeficiency (**Figure 1A**). The patient had a long history of markedly reduced social contact and limited daily social interaction before referral. Several months before rheumatology referral, he developed swelling and pain in the right elbow and visited a local orthopedic clinic. At that time, plain radiography reportedly showed soft tissue swelling without bony erosion or definite joint destruction, and the serum C-reactive protein level was elevated to 22 mg/dL. No joint aspiration or microbiological examination of synovial fluid was performed. He was diagnosed with polymyalgia rheumatica and started on oral prednisolone at 10 mg/day, although this diagnosis was not supported by the available clinical features, such as bilateral shoulder pain, hip or buttock pain, and morning stiffness were absent, and the initial manifestation was monoarticular.

A screening chest radiograph obtained at the previous clinic revealed a mass lesion in the right lower lung field, which was initially suspected to represent lung cancer. He was therefore referred to the respiratory medicine department for further evaluation. Chest computed tomography (CT) showed a mass lesion in the right lower lobe (**Figure 1B**). Sputum and bronchoalveolar lavage cultures both yielded methicillin-sensitive *Staphylococcus aureus* (MSSA). Transbronchial lung biopsy from the corresponding lesion showed no evidence of malignancy but marked neutrophilic inflammatory cell infiltration with necrotic change and little preserved normal lung tissue, consistent with lung abscess. Inpatient treatment was recommended, but the patient declined admission and was treated as an outpatient with amoxicillin/clavulanate potassium. Because follow-up CT showed insufficient shrinkage of the lesion, the initial rheumatologic diagnosis and glucocorticoid therapy were reconsidered, and he was referred to the rheumatology department.

At the time of rheumatology referral, the initial swelling and pain of the right elbow had already improved. Plain radiography of the right elbow showed no definite joint destruction (**Figure 1C**). Cutaneous examination revealed multiple dusky-red to violaceous papules and macules on the dorsal aspects of both hands and fingers, particularly around the distal and periungual regions (**Figure 1D**). Similar lesions were also present on the palmar surfaces, where they were accompanied by diffuse violaceous discoloration of the distal fingertips (**Figure 1E**). Radiographs of both hands showed soft tissue swelling of the proximal interphalangeal joints without definite bony destruction (**Figure 1F**). Musculoskeletal ultrasonography subsequently demonstrated inflammatory joint and tendon involvement. The right wrist showed severe tenosynovitis of the fourth extensor compartment (**Figure 1G**). The volar aspect of the right humeroradial joint showed mild synovial hypertrophy (**Figure 1H**). The right index metacarpophalangeal joint showed moderate synovial hypertrophy with a moderate Doppler signal and extensor peritendinous inflammation (**Figure 1I**). Additional ultrasonographic records described synovitis involving the right thumb, index, and middle metacarpophalangeal joints and the right index and middle proximal interphalangeal joints, as well as flexor tenosynovitis of the left index finger. Together, these findings supported inflammatory arthritis with synovitis and tenosynovitis in the hands and wrist, while the exact nature of the initial right elbow manifestation could not be determined retrospectively.

Laboratory findings and at rheumatology referral are summarized in **Table 1**. The patient showed profound hypogammaglobulinemia with markedly reduced circulating B cells. Rheumatoid factor (RF), anti-cyclic citrullinated peptide (anti-CCP) antibody, antinuclear antibody (ANA), myeloperoxidase-ANCA, and proteinase 3-ANCA were negative. Extended lymphocyte phenotyping was performed later in the clinical course, 15 months after the initial rheumatology referral, and is summarized in **Table 1**. Although the T-cell compartment showed a skewed CD4/CD8 ratio and a memory/activated phenotype, there was no clinical history suggestive of overt T-cell immunodeficiency, such as opportunistic fungal infection, chronic viral infection, or Pneumocystis pneumonia. These findings raised a strong suspicion of an IEI, particularly XLA. IgRT was proposed, but administrative and financial barriers delayed treatment initiation until approval of the public medical support program.

**Table 1:** Laboratory findings and lymphocyte phenotyping at rheumatology referral.

Laboratory parameter	Patient value	Reference range
Hemoglobin (g/dL)	14	10.9–15
White blood cells (×10³/µL)	3.7	3.3–8.6
MCHC (g/dL)	31.7	31–36
Hematocrit (%)	44.1	40–51
RBC (×10^6^/µL)	4.95	4.3–5.6
MCV (fL)	89.1	83–99
Platelets (×10^4^/µL)	22.4	15–35
Neutrophils (%)	22.6	38.5–80.5
Lymphocytes (%)	41.1	16.5–49.5
Monocytes (%)	21.6	2.0–10.0
Eosinophils (%)	14.2	0.0–8.5
Basophils (%)	0.5	0.0–2.5
IgG (mg/dL)	<6	861–1747
IgA (mg/dL)	3	93–393
IgM (mg/dL)	7	33–183
CRP (mg/dL)	0.48	0.00–0.14
sIL-2R (U/mL)	1892.8	156.6–474.5
RF (IU/mL)	5	0–15
C3 (mg/dL)	70	86–170
C4 (mg/dL)	4	17–45
ANA	<40	<40
Anti CCP antibody (U/mL)	<0.6	<4.5
MPO-ANCA (U/mL)	<1.0	<3.5
PR3-ANCA (U/mL)	<1.0	<3.5
Lymphocyte subset	Patient value	Reference range
T lymphocytes (CD3^+^) (% lymph.)	67.7	56.6–85.9
T-helper cells (CD3^+^CD4^+^) (% lymph.)	57.5	36.0–77.0
Cytotoxic T cells (CD3^+^CD8^+^) (% lymph.)	9.6	14.6–52.6
CD4+ helper T cells (% T cells)	84.8	–
CD8+ cytotoxic T cells (% T cells)	14.2	–
CD4/CD8 ratio	6.0	–
Memory helper T cells (% CD4+ T cells)	95.3	–
Memory cytotoxic T cells (% CD8+ T cells)	22.1	–
Activated T cells (% T cells)	92.0	–
TCR αβ T cells (% T cells)	99.2	–
TCR γδ T cells (% T cells)	0.1	–
Double-negative T cells (% αβ− T cells)	0.2	–
B lymphocytes (CD19^+^) (% lymph.)	0.0	17–41
NK cells (CD16^+^CD56^+^) (% lymph.)	23.6	0–23.5

ANA, antinuclear antibody; ANCA, anti-neutrophil cytoplasmic antibody; CCP, cyclic citrullinated peptide; CRP, C-reactive protein; lymph., lymphocytes; MCHC, mean corpuscular hemoglobin concentration; MCV, mean corpuscular volume; MPO, myeloperoxidase; PR3, proteinase 3; RBC, red blood cells; RF, rheumatoid factor; sIL-2R, soluble interleukin-2 receptor; TCR, T-cell receptor.

Before the planned initiation of IgRT, the patient developed a deep right gluteal abscess and was hospitalized. Pelvic CT showed a large abscess extending from the subcutaneous tissue into the gluteal muscle (**Figure 1J**). Incision and drainage were performed. Blood cultures were negative, whereas culture of the drained abscess yielded MSSA. Intravenous vancomycin was started empirically and then de-escalated to cefazolin based on susceptibility testing. The abscess gradually improved with treatment. During the same admission, multiple papules on the face and trunk were evaluated separately by dermatology and diagnosed as acne conglobata. Skin biopsy was not performed. These lesions improved after treatment with oral doxycycline and topical nadifloxacin.

IgRT was initiated during hospitalization and then continued regularly thereafter. Serum IgG trough levels were maintained at approximately 500–600 mg/dL. No further deep infections occurred during follow-up. Hand synovitis, tenosynovitis, and cutaneous lesions gradually improved after initiation of IgRT and treatment of documented bacterial infections.

**Figure 2:**
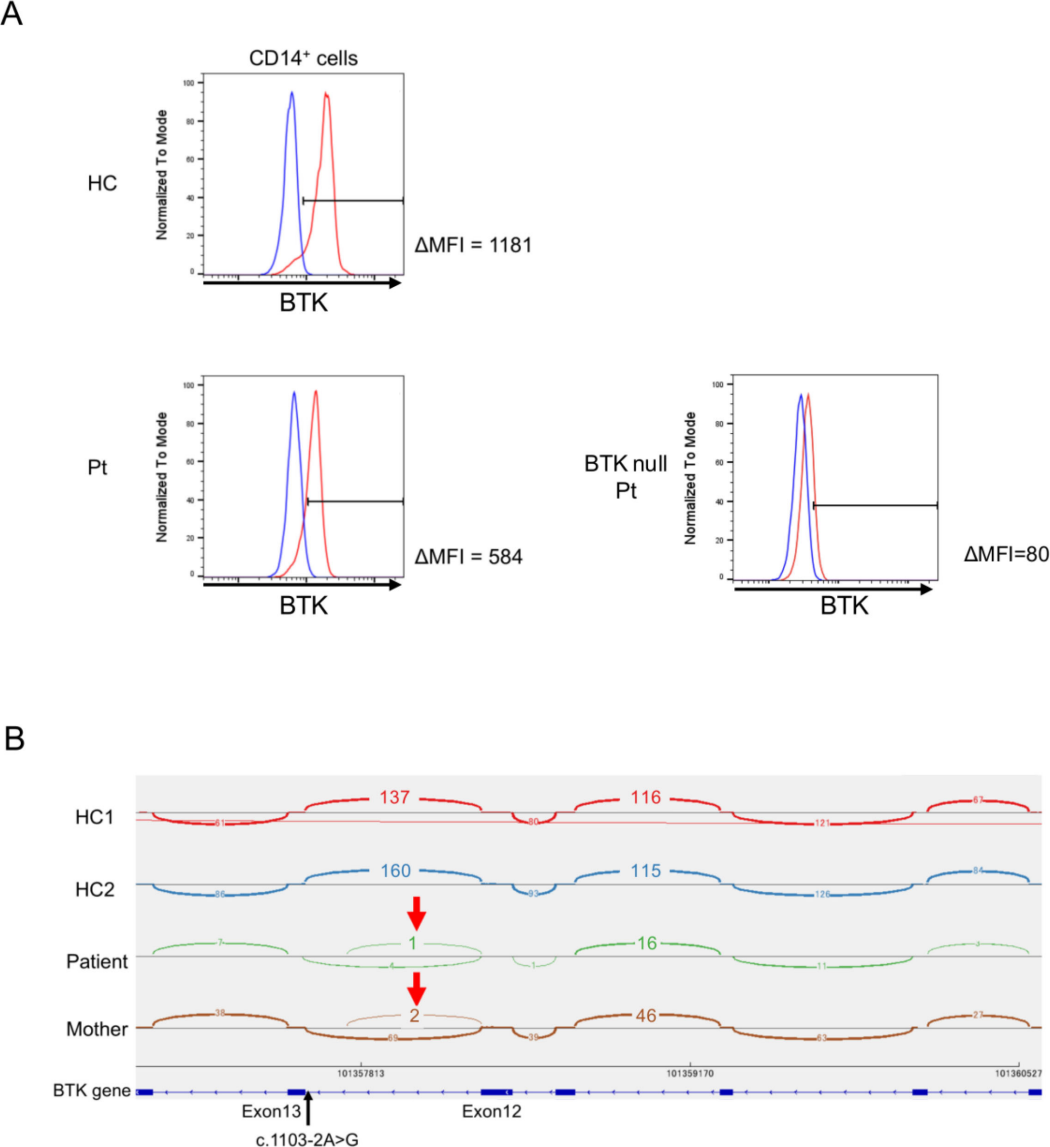
Reduced BTK protein expression and splicing abnormalities in the patient. **(A)** Histograms showing intracellular BTK staining in gated CD14^+^ monocytes from a healthy control (HC, upper panel), the patient (Pt, lower left panel), and a known BTK null XLA patient (lower right panel). The red histogram represents staining with anti-BTK antibody; the blue histogram represents the isotype control. The delta means fluorescence intensity (ΔMFI), calculated as the difference between anti-BTK and isotype control MFI values. **(B)** Sashimi plot of *BTK* RNA-seq data showing aberrant splicing at the Exon12–Exon13 junction. RNA-seq was performed on monocytes from two healthy controls (HC1, HC2), the patient, and the patient’s mother. Arc heights and overlying numbers represent splice junction read counts. In both healthy controls, only canonical splicing between Exon12 and Exon13 is observed. In the patient and his mother, aberrant splicing arcs are detected at the position of the c.1103-2A>G splice-acceptor variant (red arrows), accompanied by markedly reduced overall read counts, consistent with decreased *BTK* mRNA expression and exon skipping. Genomic coordinates on the X chromosome are shown below. HC, healthy control.

Targeted sequencing using a B-cell deficiency gene panel identified a hemizygous *BTK* splice-site variant, c.1103-2A>G (NM_000061.3). The patient’s mother was heterozygous for the same variant, consistent with carrier status (**Figure 1A**). Flow cytometric analysis of monocytes demonstrated reduced, but detectable BTK protein expression compared with healthy control cells (**Figure 2A**). Poly(A)-selected RNA sequencing further showed reduced *BTK* transcript abundance and abnormal splicing at the exon 12–13 junction, while residual normal splicing was still detectable (**Figure 2B**). These findings supported pathogenicity of the splice-site variant and were consistent with a hypomorphic or leaky phenotype.

## 3 Discussion

In this report, we identified a patient with atypical XLA who was diagnosed in adulthood during evaluation of inflammatory arthritis with ultrasonography-confirmed synovitis and tenosynovitis. The patient also developed microbiologically-documented MSSA lung abscess and deep gluteal abscess and was found to carry a *BTK* splice-site variant, c.1103-2A>G. Functional analyses demonstrated aberrant splicing, reduced *BTK* mRNA expression, and reduced but detectable BTK protein expression, consistent with a hypomorphic or leaky phenotype. Importantly, IgRT was followed by the prevention of further deep infections and the gradual improvement of the articular and cutaneous manifestations. This case provides a useful framework for discussing arthritis as a relatively common complication of XLA, the mechanisms underlying delayed diagnosis in adulthood, and the limited but clinically relevant nature of genotype-phenotype correlation in this disorder.

Arthritis is increasingly recognized as a common but underappreciated complication of XLA and may precede the diagnosis (12). In such cases, the absence of prominent recurrent infections in early life can delay recognition of XLA until adolescence or adulthood (7–11). A retrospective study of 98 XLA patients reported articular involvement in nearly half of the cohort, with pre-diagnostic joint symptoms documented in more than one-third of those affected (12). The spectrum of joint disease is broad, spanning from oligoarthritis to polyarthritis of large or small joints, frequently without erosive features, which can make the distinction from primary inflammatory arthritides challenging (12, 13). Both infectious arthritis and sterile inflammatory arthritis have been reported (15, 16). The frequent absence of identifiable pathogens and the variability of inflammatory features suggest that multiple mechanisms may contribute, including persistent infection-driven immune activation, immune complex-related inflammation, and dysregulated innate immune responses in the setting of humoral deficiency (10, 13, 17, 18). In addition, diagnosis may be challenging because, in humoral immunodeficiencies, serological markers used in the evaluation of autoimmune arthritis, including RF, anti-CCP antibodies, and ANA, may be negative or uninformative due to impaired antibody production. Accordingly, inflammatory arthritis in XLA may not be readily classified by conventional rheumatologic serology alone.

One important question is why this patient remained undiagnosed until adulthood. Our functional data showed reduced but detectable BTK protein expression in monocytes together with residual normal splicing, supporting a hypomorphic phenotype. Such residual expression may have attenuated the classic early infectious presentation. However, the delayed diagnosis is unlikely to be explained by genotype alone. Notably, the same *BTK* splice-site variant, c.1103-2A>G, was previously reported in a Malaysian cohort in twin boys who developed symptoms in early infancy and were diagnosed at 2.3 years of age, indicating that an identical variant can be associated with substantially earlier clinical presentation than in the present case (19). Previous studies have also shown that the clinical phenotype of XLA cannot always be explained by the *BTK* genotype alone, even among patients or siblings carrying the same variant (20–23). Contributing factors may include environmental exposures and immune defense mechanisms other than BTK-dependent B-cell responses. The patient had a long history of reduced social contact, which may have limited his exposure to common infectious sources and could partly explain why recurrent sinopulmonary infections were not clinically prominent. In addition, respiratory defense is determined not only by humoral immunity but also by mucosal barrier integrity, mucociliary clearance, and innate immune responses, including macrophage- and neutrophil-mediated antibacterial defense (24–26). We therefore interpret the delayed diagnosis in this patient as the result of interacting genetic, environmental, and host defense factors, rather than as a phenomenon determined solely by *BTK* genotype.

This interpretation is consistent with the limited literature specifically addressing genotype-phenotype correlation in XLA (**Table 2**). Among studies designed for this question, López-Granados et al. (21) directly examined BTK genotype, protein expression/function, and clinical phenotype in 54 patients and concluded that genotype-phenotype correlation exists but is incomplete. Subsequent genotype-focused cohort studies by Broides et al. (22), Teimourian et al. (27), and Lee et al. (28) likewise supported only a limited, non-deterministic relationship, in which less disruptive or hypomorphic variants tended to be associated with older age at diagnosis, higher serum IgM, or slightly more residual peripheral B cells, whereas more disruptive variants tended to present earlier and with more severe infections. Case-based reports are compatible with this interpretation: a kinase-domain missense variant produced a mild “leaky” phenotype in three affected brothers with detectable immunoglobulins and low but not absent B cells (29), and a minimally hypomorphic BTK variant reduced B-cell numbers and BTK signaling without overt clinical disease (30). However, deterministic correlation is not supported across the broader literature. Kanegane et al. (23) documented marked clinical heterogeneity, including delayed diagnosis and atypical phenotypes, and family-based studies showed substantial phenotypic discordance despite shared BTK variants, both in affected siblings (20) and in a three-generation family (31). Taken together, the available evidence supports a genotype-phenotype correlation in XLA that is present but limited: residual BTK expression or partial function may attenuate the classical phenotype, but genotype alone does not reliably predict individual disease severity.

**Table 2:** PubMed-indexed studies relevant to genotype-phenotype correlation and phenotypic heterogeneity in X-linked agammaglobulinemia.

Study	Design/Numbers of Pt	Main finding
López-Granados et al., 2005 (21)	Cohort study/54 patients with XLA	Genotype-phenotype correlation was present but incomplete; less disruptive variants tended to be associated with older age at diagnosis, higher serum IgM, and slightly more residual peripheral B cells.
Broides et al., 2006 (22)	Cohort study/110 patients with XLA from 94 unrelated families	Mutation-specific effects were detectable, but no clear deterministic genotype-phenotype rule was established across XLA families.
Teimourian et al., 2008 (27)	Cohort study	In a genotype-focused cohort, severe genotypes did not necessarily lead to severe phenotypes, supporting a limited and non-deterministic genotype-phenotype relationship.
Lee et al., 2010 (28)	Cohort study/62 patients with XLA	More disruptive *BTK* variants were associated with earlier disease onset and more severe infectious phenotypes, supporting a weak but non-deterministic genotype-phenotype correlation.
Jones et al., 1996 (29)	Case report	Three affected brothers carrying a BTK kinase-domain missense variant showed a mild “leaky” XLA phenotype with detectable immunoglobulins, some specific antibody production, and low but not absent B cells, suggesting that partial preservation of BTK function may attenuate the classical phenotype.
Conley et al., 2008 (30)	Case report with mechanistic study	A minimally hypomorphic BTK mutation (Y418H) can reduce peripheral B-cell numbers and mildly impair Btk signaling, yet may be insufficient by itself to cause overt clinical immunodeficiency; therefore, a BTK mutation plus reduced B cells does not always predict clinical disease.
Kanegane et al., 2001 (23)	Cohort study/95 patients with XLA	No clear deterministic genotype–phenotype correlation was established in XLA; however, a substantial subset of patients showed delayed diagnosis and relatively preserved serum IgG, suggesting that milder or atypical phenotypes can occur despite *BTK* variants.
Bykowsky et al., 1996 (20)	Sibling report	Siblings carrying the same *BTK* initiation-codon variant showed discordant clinical phenotypes, providing direct evidence that factors beyond the *BTK* genotype influence disease expression.
Kornfeld et al., 1997 (31)	Three-generation family report	Marked intrafamilial phenotypic variation was observed despite the same *BTK* nonsense variant, with manifestations ranging from mild to severe and extending to diagnosis in adulthood.
Chear et al., 2023 (19)	Cohort study/22 patients with XLA	The same *BTK* c.1103-2A>G splice-site variant was identified in twin boys with symptom onset in early infancy and diagnosis at 2.3 years of age, indicating that the identical variant can be associated with substantially earlier and more overt clinical presentation than in the present case.
Carrillo-Tapia et al., 2018 (9)	Genotype-focused clinical study/5 atypical XLA patients with delayed diagnosis	Delayed diagnosis was enriched among patients with non-kinase-domain variants and detectable BTK expression, suggesting that residual BTK expression may attenuate the classical early phenotype, although genotype alone did not predict clinical severity.

In conclusion, this case illustrates that atypical XLA may remain unrecognized until adulthood and present with common rheumatologic manifestations, including arthritis, synovitis, and tenosynovitis. Although the *BTK* splice-site variant identified in this patient was associated with residual protein and transcript expression consistent with a hypomorphic or leaky phenotype, the delayed clinical presentation was likely shaped not by genotype alone but by interacting host and environmental factors. The favorable response to IgRT, with prevention of further deep infections and gradual improvement of articular and cutaneous manifestations, further supports the importance of timely restoration of humoral immunity. For adult patients presenting with inflammatory joint disease, especially when accompanied by hypogammaglobulinemia, reduced B cells, chronic lung lesions, or deep bacterial abscesses, clinicians should consider the possibility of an underlying IEI such as XLA. This case also highlights the value of integrating immunologic evaluation, genetic testing, and detailed protein- and transcript-level analyses when interpreting atypical presentations and genetic variants.

## 4 Materials and methods

### Human subjects

All enrolled subjects provided written informed consent and were collected through protocols following local ethics and IRB approval at Chiba University Hospital (HS202207-05).

### Gene panel sequencing analysis

Targeted gene panel sequencing for B-cell deficiency disorders was performed using a diagnostic gene panel provided by Kazusa DNA Research Institute.

### Flow cytometric analysis of BTK protein

Peripheral blood mononuclear cells (PBMCs) were stained with phycoerythrin-conjugated anti-CD14 monoclonal antibody (mAb) (Dako). Cells were fixed with 4% paraformaldehyde and are permeabilized with 0.1% triton X. Cells are incubated with anti-BTK (OriGene) or isotype mAb and reacted with fluorescein isothiocyanate-conjugated anti-mouse IgG2a (eBioscience). Stained cells were analyzed by flow cytometry.

### RNA sequencing

Peripheral blood mononuclear cells (PBMCs) were collected from the patient, the patient’s mother, and two healthy male controls. RNA sequencing was performed at the Kazusa DNA Research Institute using the NEBNext Ultra II Directional RNA Library Prep Kit for Illumina (NEB) with poly(A) selection for mRNA enrichment, followed by sequencing on an Illumina NextSeq500 platform. Gene expression analysis was performed using Strand NGS, and sashimi plots for the BTK gene were generated using the Integrative Genomics Viewer (IGV).

### Focused literature review of *BTK* genotype–phenotype correlation in X-linked agammaglobulinemia

A focused literature review was performed to contextualize the present case within the published evidence on *BTK* genotype–phenotype correlation in XLA. PubMed was searched on April 20, 2026, using the terms “X-linked agammaglobulinemia,” “XLA,” “BTK,” “Bruton tyrosine kinase,” and “genotype-phenotype correlation” or related phenotype terms. Original cohort studies, family reports, and case reports were reviewed when BTK genotype and clinically interpretable phenotype, including age at onset or diagnosis, residual BTK expression or function, and atypical or delayed presentation, could be extracted. Reports lacking sufficient phenotype information were excluded.

## Data Availability

The datasets generated and analyzed during the current study are not publicly available because they contain identifiable patient genetic information that could compromise participant privacy. De-identified data are available from the corresponding author on reasonable request, subject to approval by the Chiba University Hospital ethics committee and a data-sharing agreement.
