# Supplementary material for: Atypical X-linked agammaglobulinemia diagnosed in adulthood with arthritis caused by a hypomorphic BTK splice-site variant: a case report and literature review
Source: Front Immunol. 2026 May 21;17:1849090. doi: 10.3389/fimmu.2026.1849090 (PMC13233687; doi:10.3389/fimmu.2026.1849090)
Supplement: Supplementary file 1 [file Table1.docx]

**CARE Checklist**

Information to include when writing a case report

*Takayama A, Meguro K, et al. – Adult-onset X-linked agammaglobulinemia diagnosed following inflammatory arthritis*

| **Topic** | **Item #** | **Checklist item description** | **Reported on Line #** | **Where reported** |
| --- | --- | --- | --- | --- |
| **Title** | **1** | The diagnosis or intervention of primary focus followed by the words “case report” | Yes | Title page |
| **Key Words** | **2** | 2 to 5 key words that identify diagnoses or interventions in this case report, including “case report” | Yes | Title page |
| **Abstract (no references)** | **3a** | Introduction—What is unique about this case and what does it add to the scientific literature? | Yes | Abstract, para. 1 |
|  | **3b** | Main symptoms and/or important clinical findings | Yes | Abstract, para. 2 |
|  | **3c** | The main diagnoses, therapeutic interventions, and outcomes | Yes | Abstract, para. 2 |
|  | **3d** | Conclusion—What is the main “take-away” lesson(s) from this case? | Yes | Abstract, para. 3 |
| **Introduction** | **4** | One or two paragraphs summarizing why this case is unique (may include references) | Yes | Introduction |
| **Patient Information** | **5a** | De-identified patient specific information | Yes | Case Presentation, para. 1 |
|  | **5b** | Primary concerns and symptoms of the patient | Yes | Case Presentation, para. 2–3 |
|  | **5c** | Medical, family, and psycho-social history including relevant genetic information | Yes | Case Presentation, para. 1; Fig. 1A |
|  | **5d** | Relevant past interventions with outcomes | Yes | Case Presentation, para. 2–3 |
| **Clinical Findings** | **6** | Describe significant physical examination (PE) and important clinical findings | Yes | Case Presentation, para. 3; Tables 1–2; Fig. 1C–E |
| **Timeline** | **7** | Historical and current information from this episode of care organized as a timeline | Yes | Case Presentation, para. 1–5 |
| **Diagnostic Assessment** | **8a** | Diagnostic testing (such as PE, laboratory testing, imaging, surveys) | Yes | Case Presentation, para. 3–5; Tables 1–2; Fig. 1–2 |
|  | **8b** | Diagnostic challenges (such as access to testing, financial, or cultural) | Yes | Case Presentation, para. 2–3; Discussion, para. 1–2 |
|  | **8c** | Diagnosis (including other diagnoses considered) | Yes | Case Presentation, para. 2, 5 |
|  | **8d** | Prognosis (such as staging in oncology) where applicable | N/A | N/A |
| **Therapeutic Intervention** | **9a** | Types of therapeutic intervention (such as pharmacologic, surgical, preventive, self-care) | Yes | Case Presentation, para. 2, 4 |
|  | **9b** | Administration of therapeutic intervention (such as dosage, strength, duration) | Yes | Case Presentation, para. 2, 4 |
|  | **9c** | Changes in therapeutic intervention (with rationale) | Yes | Case Presentation, para. 2–4 |
| **Follow-up and Outcomes** | **10a** | Clinician and patient-assessed outcomes (if available) | Yes | Case Presentation, para. 4 |
|  | **10b** | Important follow-up diagnostic and other test results | Yes | Case Presentation, para. 5; Fig. 2A–B |
|  | **10c** | Intervention adherence and tolerability (How was this assessed?) | Yes | Case Presentation, para. 4 |
|  | **10d** | Adverse and unanticipated events | Yes | Case Presentation, para. 4 |
| **Discussion** | **11a** | A scientific discussion of the strengths AND limitations associated with this case report | Yes | Discussion |
|  | **11b** | Discussion of the relevant medical literature with references | Yes | Discussion |
|  | **11c** | The scientific rationale for any conclusions (including assessment of possible causes) | Yes | Discussion |
|  | **11d** | The primary “take-away” lessons of this case report (without references) in a one paragraph conclusion | Yes | Discussion, final para. |
| **Patient Perspective** | **12** | The patient should share their perspective in one to two paragraphs on the treatment(s) they received | N/A | N/A |
| **Informed Consent** | **13** | Did the patient give informed consent? Please provide if requested | Yes | Material and methods |

**Informed Consent:** Written informed consent was obtained from the patient (IRB approval: HS202207-05, Chiba University Hospital).
